# Delving into the relationship between teacher emotion regulation, self-efficacy, engagement, and anger: A focus on English as a foreign language teachers

**DOI:** 10.3389/fpsyg.2022.1019984

**Published:** 2022-10-19

**Authors:** Juan Deng, Tahereh Heydarnejad, Fariba Farhangi, Ayman Farid Khafaga

**Affiliations:** ^1^School of Foreign Languages, Huanggang Normal University, Huanggang, China; ^2^Department of English Language, Faculty of Literature and Humanities, University of Gonabad, Gonabad, Iran; ^3^Department of English Language and Literature, Khazar University, Baku, Azerbaijan; ^4^Department of English, College of Science and Humanities, Prince Sattam Bin Abdulaziz University, Al-Kharj, Saudi Arabia; ^5^Department of English, Faculty of Arts and Humanities, Suez Canal University, Ismailia, Egypt

**Keywords:** teacher emotion regulation, teacher self-efficacy beliefs, teacher engagement, teacher anger, EFL teachers

## Abstract

Due to the potent role of teachers’ emotion regulation in effective teaching, it seems essential to see how emotion regulation can contribute to other relevant teaching constructs. In this regard, the present study is intended to probe into the causal relationship among teacher emotion regulation, self-efficacy beliefs, engagement, and anger. In so doing, the Language Teacher Emotion Regulation Inventory (LTERI), The Teacher Sense of Efficacy Scale (TSES), The Engaged Teacher Scale (ETS), and The Teacher Anger Scale (TAS) were administered to 581 English as a Foreign Language (EFL) teachers in Iran. To gauge the causal relationships among the variables, confirmatory factor analysis (CFA) and structural equation modeling (SEM) using LISREL 8.80 were conducted. The results indicated that language teacher emotion regulation could positively and significantly predict teachers’ self-efficacy beliefs and engagement at work. Moreover, the influence of language teacher emotion regulation on the teacher’s anger is significantly negative. That is, the stronger emotion regulation is implemented the better teachers can manage their anger. The implications of this study may uncover new prospects for effective teaching, especially during the COVID-19 pandemic.

## Introduction

Emotions are an inevitable part of teaching. Considering the indisputable relevance of emotions experienced in the workplace for teachers’ effectiveness, it is important that teachers employ effective strategies in order to regulate such emotions. Through the lens of emotion regulation, teachers are able to evaluate and modify the intensity and duration of the emotional experiences in the workplace (Chang and Taxer, 2021). Moreover, due to the potent role of teachers’ emotion regulation in effective teaching, it seems essential to see how emotion regulation can contribute to other relevant teaching constructs.

Self-efficacy as a prominent teacher-related construct affects every area of individuals’ endeavors. As voiced by Bandura (1997), self-efficacy is the individuals’ evaluation of their potential to adjust and structure activities to achieve their ultimate objectives. In the realm of teaching, self-efficacy beliefs shape how teachers approach goals, challenges, and class activities (Lazarides and Warner, 2020; Liu F. et al., 2021). In Chen’s words (2018), efficacious and positive teachers are more successful. Efficacious teachers show more job satisfaction and deal with students’ misbehavior and demotivation more efficiently (Burić and Kim, 2020; Vadivel et al., 2021). In contrast, teachers with a low sense of self-efficacy are apt to lose faith in their abilities (Barni et al., 2019). The research history on teacher self-efficacy shines back on the reciprocal relationships between self-efficacy and an array of positive teacher and student-related issues (Martin and Mulvihill, 2019; Amirian et al., 2022; Ma, 2022; Zheng et al., 2022).

In recent years, studies on the associations between teacher self-efficacy and work engagement have been quite rosy (Li et al., 2019; Zeng, 2020; Rezai et al., 2022). For instance, recent studies indicate that teachers with higher perceived self-efficacy are more engaged in their work activities (Burić and Macuka, 2017; Li et al., 2019; Xu et al., 2022). Engaged teachers show high levels of dedication and commitment to educational objectives; they are completely immersed in the class activities and attentive to their students’ needs (Klassen et al., 2013; Burić and Macuka, 2017; Addimando, 2019; Vadivel and Beena, 2019). Today’s continuously challenging environment, in particular, the current outbreak of the COVID-19 pandemic, calls for engaged teachers who regulate their emotions. Among the primary emotions of human beings, anger is repeatedly used, and it is one of the six emotions with noticeable facial expressions across cultures, along with fear, sadness, surprise, disgust, and joy (Ekman, 1992; Namaziandost and Çakmak, 2020).

Based on the existing literature on teachers’ emotions, the most prominent experienced positive emotion is happiness, while anger is the most prominent experienced negative emotion in teachers’ professional lives (Chang, 2013; Frenzel, 2014; Burić and Frenzel, 2019; Azizi et al., 2022). The main potential stimuli triggering teachers’ anger in their professional lives are disciplinary issues and lack of student commitment to tasks and homework (Khajavy et al., 2018), uncooperative colleagues and parents’ misbehavior (Sutton and Wheatley, 2003), blocked academic goals and students’ inappropriate manners (Sutton, 2007), as well as educational policies and school organization (Burić and Frenzel, 2019). Other potential sources of teacher anger include the COVID-19 pandemic and lockdown, as well as remote online teaching and uncertainty about the future. Based on the existing literature on teacher’s anger, it is positively correlated with teachers’ burnout (Chang, 2013; Tilwani et al., 2022) and emotional exhaustion (Burić and Moè, 2020). In contrast, the interplay between teachers’ anger and their job satisfaction (Burić and Frenzel, 2019), wellbeing, and self-efficacy beliefs is negative (Burić and Kim, 2020). In response to the challenges of the twenty-first century, arming teachers with self-aid constructs that target effective teaching should be considered at the forefront of education policies. Language teachers and university professors are by no means an exception, and their significant role in effective language teaching cannot be overlooked.

**FIGURE 1:**
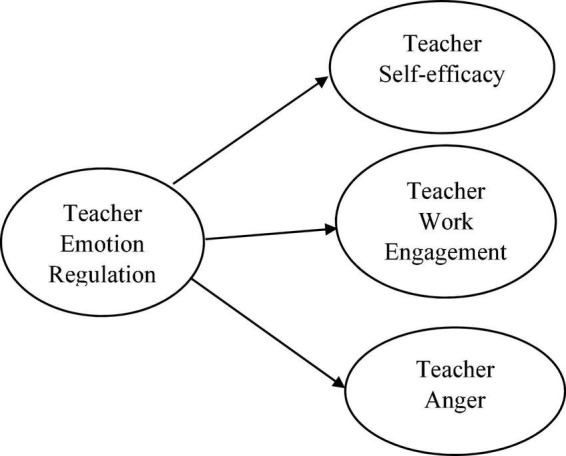
Theoretical structural equation model.

What emerges from the review of the related literature is that the associations among language teacher emotion regulation, perceived self-efficacy beliefs, work engagement, and anger have not been brought to the foreground of research foci (Gross and Thompson, 2007; Burić et al., 2017; Abdollahi et al., 2022), particularly in the EFL context (Chahkandi et al., 2016; Fathi and Derakhshan, 2019; Chang, 2020; Chang and Taxer, 2021; Li and Liu, 2021; Liu F. et al., 2021). Therefore, the dearth of research in this domain calls for more studies that put forward a clear picture of the influence of emotion regulation on teachers’ sense of self-efficacy, work engagement, and anger. More precisely, how emotional regulation serves as underlying principles that form teachers’ efficacy beliefs, work engagement, and anger management in the context of Iran, where English is taught as a foreign language. Thus, to delve into the construct of language teacher emotion regulation in the Iranian EFL context, the present study puts forward the need to examine the association of language teachers’ emotion regulation with their efficacy beliefs, work engagement, as well as anger (see Figure 1). The outcome of such an exploration is significant in the teacher’s effectiveness as it could illuminate how emotion regulation as an important skill empowers language teachers to modify and manage themselves efficaciously and to be engaged even in emotion-provoking situations at the workplace. This study set out to fill in this educational gap by answering the following research questions:

1. To what extent could EFL teachers’ emotion regulation predict their self-efficacy beliefs in the workplace?
2. To what extent could EFL teachers’ emotion regulation predict their engagement in the workplace?
3. To what extent could EFL teachers’ emotion regulation predict their anger in the workplace?

In this regard, the following null hypotheses could be formulated:

HO1. EFL teachers’ emotion regulation has no impact on their self-efficacy beliefs in the workplace.

HO2. EFL teachers’ emotion regulation has no impact on their engagement in the workplace.

HO3. EFL teachers’ emotion regulation has no impact on their anger in the workplace.

### Literature review

In the following sections, an overview of the relevant literature on emotion regulation, self-efficacy, work engagement, and anger is provided.

### Emotion regulation

Emotion regulation can be defined as physiological, behavioral, and cognitive processes that individuals employ to effectively manage and respond to emotional experiences (Gross, 1998; Gross and John, 2003; Gross and Thompson, 2007). That is, emotion regulation shapes which emotions people have, when they have them, and how they experience or express them (Gross, 1998; Kolganov et al., 2022). Similar to other types of self-regulation and self-management skills, experienced emotions are modulated to fulfill educational objectives; and, to do so, various strategies may be applied, which are defined as emotion regulation strategies (Taxer and Gross, 2018; Taylor et al., 2020; Alipour et al., 2021; Li and Liu, 2021). In the classroom, both teachers and students are exposed to different emotional experiences. The teachers, as the center of the classroom are expected to establish an optimal emotional climate; they should manage their own emotions and their learners’ emotions (Taxer and Gross, 2018). Simply put, emotion regulation empowers both teachers and students to strengthen pleasant emotions and weaken unpleasant emotions (Fried, 2011).

Previous studies in the realm of emotion regulation have documented the predominance and significant contributions of teacher emotion regulation to effective teaching. For instance, Taxer and Gross (2018) studied the aim and quality of emotion regulation in classes. They concluded that teachers with instrumental and hedonic emotion regulation goals tried to manage their own and their learners’ emotions. Similarly, Morris and King (2018) investigated the effective role of emotion regulatory strategies in managing the in-class frustration experienced by university language teachers. According to their findings, language university teachers employed contextually dependent emotion regulation behaviors that help them to increase levels of confidence and control over the stressors.

In a recent study by Chang and Taxer (2021), teacher emotion regulation strategies in response to classroom misbehavior were investigated. They concluded that teachers who usually reappraise are less likely to experience unpleasant emotions in the face of students’ misbehavior and express fewer suppression experiences when negative emotions are felt. The implemented strategies to regulate emotions were classified as reappraisal or suppression. The researchers of this study suggested further study to inspect the effectiveness of other strategies used to regulate emotions. In the same token, Chang (2020) explored the association between teachers’ beliefs about emotional display rules in the class, their attitudes toward emotion regulation strategies, and feelings of burnout. This study concluded that display rules affect expressive suppression and burnout. Likewise, Morris and King (2018) conducted a series of semi-structured interviews, classroom observations, and corresponding stimulated-recall sessions among seven EFL teachers at a university in Japan. Based on the data analysis, a major mechanism for reducing the experienced frustrations among EFL university teachers is emotion regulation.

In light of the undeniable importance that teachers’ emotions and emotion regulation have for effective teaching and learning, Jiang et al. (2016) carried out a mixed-method study. In so doing, the students’ perceptions of their teachers’ emotions and emotion regulation while teaching were explored. The results of the students’ survey and teachers’ interviews indicated that antecedent-focused emotion regulation appeared more preferable than response-focused emotion regulation. Moreover, reappraisal was more effective than suppression in increasing positive-emotion expression and decreasing negative-emotion expression. In like manner, Fathi et al. (2021) explored a structural model of teacher reflection, self-efficacy, burnout, and emotion regulation among Iranian EFL instructors. They found that teacher self-efficacy and reflection were positively correlated with emotion regulation. Also, their findings revealed that teacher emotion regulation was negatively correlated with burnout.

### Self-efficacy

The concept of self-efficacy, rooted in Albert Bandura’s social-cognitive theory of behavioral change, is conceptualized as individuals’ confidence in their abilities to implement the courses of action necessary to produce a given accomplishment (Bandura, 1997, 1986). This definition highlights that people with higher degrees of confidence in their capabilities could have greater chances of achieving the desired outcomes due mainly to a clearer sense of purpose and a stronger will to surmount the obstacles in a given task. Bandura (1986) believed this metacognitive capability is manifested through self-regulatory processes, which connect thought to action and include self-monitoring, performing self-guidance through personal standards, and corrective self-reactions. Bandura’s agentic socio-cognitive theory (1986) also emphasized the individual’s ability to reflect on themselves, their thoughts, and actions.

Self-efficacy mirrored individuals’ potential to organize and perform the requirements of an action to fulfill an achievement (Bandura, 1997). It affects individuals’ thinking, their future actions, their coping strategies while facing emotional demands, and the attempts they put forth in a given endeavor (Bandura, 2012). Self-efficacy is both a personal and social construct because each person functions individually and collectively. Individuals’ concerns about their collective efficacy influence what they choose to do as a group, how much attempt they make to perform it, their persistence and tolerance, and their probability of success (Bandura, 1997; Schunk and Pajares, 2002).

Self-efficacy is one of the important factors that influence adjusting teaching tone, which leads to beneficial outcomes in learning and cyclically increases teacher efficacy. This specific form of efficacy is known as teacher efficacy. Simply put, teachers’ faith in their ability can influence their attitude, commitment, motivation, willingness, and effectiveness (Barni et al., 2019) and support students’ outcomes (Martin and Mulvihill, 2019). Efficacy beliefs influence teachers’ instructional practices, inter- and intra-teacher relationships (Martin and Mulvihill, 2019), and their passion for teaching (Moè, 2016). Furthermore, teachers’ efficacy is associated with learners’ motivation, achievement, and efficacy (Tschannen-Moran et al., 1998). High-efficacy teachers experience lower stress levels and less burnout than low-efficacy teachers (Chen, 2018). It is noteworthy that academic and theoretical knowledge of language teaching by no means guarantees a teacher’s level of self-efficacy.

In a recent study by Burić and Kim (2020), the positive effect of teacher self-efficacy on three aspects of instructional quality, namely classroom management, cognitive activation, and supportive climate, was concluded. Furthermore, the negative relationships between teacher self-efficacy and anger were supported by Burić and Frenzel (2019). In another recent investigation, the predictive impact of teachers’ work engagement on their self-efficacy was determined (Li et al., 2019). In the same line of inquiry, the role of teachers’ motivations in determining the strength of the relationships between teacher self-efficacy, openness to change, and self-transcendence was endorsed (Barni et al., 2019).

In an attempt to further clarify the predictive role of EFL teachers’ self-efficacy and collective teacher efficacy in affecting the psychological wellbeing in the Iranian context, a survey was conducted by Fathi et al. (2020), in which they concluded that teacher self-efficacy was a stronger predictor of psychological wellbeing than collective teacher efficacy. Likewise, Zheng et al. (2022) investigated the contributions of self-efficacy and emotion regulation to L2 teacher grit. Their findings suggest that self-efficacy and emotion regulation are major determinants of university professors’ L2 grit. Furthermore, the results of this study highlighted the critical role of self-efficacy beliefs in fostering emotion regulation among university professors. Taking a similar path, Li et al. (2022) concluded that emotion regulation and critical thinking could predict immunity in higher education.

### Work engagement

Work engagement offers a royal road to professional development. This is an affective-motivational construct with an emphasis on willingness and involvement at work (Silva et al., 2020). It is generally considered to be associated with job involvement (Lawler and Hall, 1970), job commitment (Mowday, 1998), and job empowerment (Thomas and Velthouse, 1990). The first conceptualization of work engagement originated from Kahn (1990), who defined this concept as the absorbing emotionally, cognitively, and physically into one’s job responsibilities. As an affective-motivational construct, work engagement reflects individuals’ desire for involvement in and gratification in the job (Van Beek et al., 2011). In other words, work engagement is the allotment of an individual’s resources to do their best at work (Christian et al., 2011; Wang et al., 2022). Schaufeli and Salanova (2011) viewed work engagement as a work-related state of mind established by vigor, dedication, and absorption. Vigor is physical or mental energy and determination to do work activities. Dedication refers to one’s willingness and enthusiasm for his/her work. Absorption means completely engrossed and preoccupied with one’s work (Schaufeli and Bakker, 2004).

Different variables influence how individuals perceive themselves, their work, and the relationships between the two. As Kahn (1990) stipulated, individuals present varying degrees of their selves, physically, cognitively, and emotionally, in the roles they perform. In this regard, Kahn (1990) utilized the concept of self-in-role and assumed that individuals show different depths of their selves while performing different activities during their work days.

Due to different conceptualizations and various definitions of the concept of work engagement, inconsistencies developed in the way it was assessed (Schaufeli and Salanova, 2011; Klassen et al., 2013; Kuok and Taormina, 2017; Shu, 2022). In this study, the Engaged Teacher Scale (ETS) was utilized, which was developed and validated by Klassen et al. (2013). ETS is a multi-dimensional measure of work engagement that is specifically targeted at the work carried out by teachers in classrooms and schools. Cognitive-physical engagement, emotional engagement, social engagement among students, and social engagement among colleagues are the four components of this scale. Cognitive-physical engagement is the extent to which teachers cognitively and physically devote their time, energy, and attention to teaching tasks. Emotional engagement refers to teachers’ positive emotional responses to different activities at work. The distinguishing feature of this model is that it adds a dimension for assessing social engagement (energy devoted to establishing relationships) and its causal explanations among teachers. Recent studies by Silva et al. (2020), Topchyan and Woehler (2020), and Shu (2022) confirmed and supported the good psychometric qualities of the ETS in educational settings.

Teaching is influenced by teachers’ social relationships with students and colleagues, and these reciprocal relationships play mediating roles in enhancing student engagement and positive student outcomes (Oga-Baldwin and Nakata, 2020). Effective teachers are those who are socially engaged with their students (Jennings and Greenberg, 2009; Wang et al., 2022). Research on teacher work engagement seems to be scarce. However, the previous studies on teacher work engagement highlighted teacher-related variables that affect or are affected by work engagement. To be more specific, the existing literature highlighted the close relationships between work engagement and other positive teacher-related outcomes. For instance, Zeng et al. (2019) examined the interplay among growth mindset, work engagement, perseverance of effort, and wellbeing for teachers in China. The data analysis indicated that growth mindset, wellbeing, and perseverance of effort could all predict work engagement. In another study by Oga-Baldwin and Nakata (2020), the influence of engaging teacher practices was explored, and their results indicated that students are more responsive and active in classes with engaged teachers.

Likewise, Topchyan and Woehler (2020) investigated the effects of teacher status (full-time vs. substitute) and its interaction with demographic information on the teacher’s job satisfaction and work engagement. Based on the findings of this survey, both status and gender influence teachers’ job satisfaction and work engagement. Furthermore, female teachers were more engaged with students and the length of teaching experience did not affect job satisfaction or work engagement. In a cross-contextual analysis, the role of emotion regulation and psychological wellbeing as predictors of work engagement was explored (Greenier et al., 2021), and concluded that emotion regulation and psychological wellbeing lead to work engagement among British and Iranian teachers. Nevertheless, psychological wellbeing displayed a stronger prediction of work engagement than emotion regulation. Also, it was found out that the association between psychological wellbeing and work engagement was stronger for British teachers.

### Anger

Anger is an instinctive reaction to circumstances where a person feels offended or wronged, which is usually activated by external factors such as injustice, humiliation, physical conditions or illnesses, etc. (Harmon-Jones and Harmon-Jones, 2016). Anger physiologically causes the release of stress hormones, increases heart rate, blood pressure, heavy breathing, and body temperature (Hall, 1899). The review of the literature on anger conceptualizations demonstrated that, for centuries, anger was regarded as a sin, a weakness, or as a madness; thus, it was to be avoided or controlled (Thomas, 1990). Then, the Western view of anger began to change, partly due to the misinterpretation of Freud’s work (Thomas, 1990). Furthermore, this shift of interpretation is owed to Darwinian evolutionary theory and the research of ethologists such as Konrad Lorenz (Thomas, 1990). Following this stage, Americans came to believe that it is not always possible to control this powerful emotion. This standpoint may account for a sharp increase in violent crimes in America in comparison to Japan, where people believe anger should be controlled (Harmon-Jones and Harmon-Jones, 2016). However, the theory of innate aggression was rejected because there was no scientific basis to support the notion that humans are instinctively aggressive (Kuppens et al., 2003).

According to Burić and Frenzel (2019), teacher anger, like other human emotions, is a complex and multifaceted construct, consisting of various distinguishable emotional components (subjective, cognitive, motivational, expressive, and physiological). Among the potential stimuli triggering teachers’ anger at work could be uncooperative colleagues, parents’ misbehavior (Sutton and Wheatley, 2003), blocked academic goals and students’ inappropriate manners (Sutton, 2007), as well as educational policies and school organization (Burić and Frenzel, 2019). Thus, teacher anger is triggered not only because of their students, but also because of their colleagues, students’ parents, and the educational system (Burić and Frenzel, 2019). Furthermore, Jacob et al. (2017) asserted, based on the reciprocal model on the causes and effects of teacher emotions, that situations and events that teachers perceive as impeding their goals, as well as appraisals of responsibility for this goal impediment, trigger their anger.

An overview of the literature on teacher anger shows that it is positively correlated with teachers’ burnout (Chang, 2009; Liu and Chu, 2022), surface acting (Taxer and Frenzel, 2015), and emotional exhaustion (Chang, 2013; Burić and Moè, 2020). By contrast, the relationships between teacher anger and their wellbeing (Burić et al., 2020) as well as job satisfaction (Macdonald, 1999) are proved to be negative. In a similar vein, the relationships among teacher anger, emotional labor, wellbeing, and teachers’ self-efficacy were examined by Burić and Frenzel (2019). The data analysis indicated that all facets of teacher anger were positively correlated with surface acting and deep acting was unrelated to teacher anger. Moreover, teachers who attempt more to really experience and show the expected emotions tend to experience less anger in relation to their students.

### Theoretical framework

To explain emotion regulation, the process-oriented model of emotion regulation with five temporal points (i.e., situation selection, situation modification, attention deployment, cognitive change, and response modulation) is proposed by Gross (1998). By extensive review of the existing literature, the theoretical conceptualizations on the emotion regulation in general, and teacher emotion regulation in particular, a new model for language teacher emotion regulation was introduced by Heydarnejad et al. (2021b). This model includes six dimensions, i.e., situation selection, situation modification, attention deployment, reappraisal, suppression, and seeking social support. The three dimensions of situation selection, situation modification, and attention deployment were rooted in Gross’s process-oriented model of emotion regulation (1998). Reappraisal and suppression were based on Gross and John’s conceptualization (2003), and seeking social support as the last dimension was inspired by Jennings and Greenberg (2009) as well as Taxer and Gross (2018).

In the teaching context, Bandura’s self-efficacy theory reflected teachers’ beliefs about their abilities to engage their learners effectively and to achieve educational objectives efficiently (Tschannen-Moran et al., 1998; Heydarnejad et al., 2021a; Sudina et al., 2021). Bandura (1997) introduced sources of efficacy beliefs as mastery experience, vicarious experience, social or verbal persuasion, and physiological or affective states. Among these factors, mastery experience has the most influential role in self-efficacy beliefs. The next step is observing a successful performance by other similar people (Tompson and Dass, 2000). The third source of self-efficacy is originated from social or verbal persuasion. Successful persuasion fosters people’s perceptions of their abilities and future accomplishments (Schunk and Pajares, 2002). Psychological and affective states as the last source highlight the role of positive factors in boosting individuals’ efficacy beliefs (Bandura, 1997).

The concept of work engagement is theoretically supported by self-determination theory (SDT) (Bakker et al., 2011; Zeng et al., 2019). As SDT suggests, individuals who are engaged in their work are encouraged to bring improved presentation, perseverance, and inventiveness to their work (Oga-Baldwin and Nakata, 2020). To define teacher engagement, Klassen et al. (2013) developed a model including three dimensions: cognitive-physical engagement, emotional engagement, social engagement with students, and social engagement with colleagues. Cognitive-physical engagement is the cognitive and physical devotion of teachers to their teaching. Emotional engagement refers to teachers’ enjoyment and entertainment aligned with instruction (Han and Wang, 2021). Social engagement with students and social engagement with colleagues are the last two dimensions of this model, which focus on the social dimension of teachers’ jobs (Jennings and Greenberg, 2009; Oga-Baldwin and Nakata, 2020).

Various schools of thought conceptualize anger from different perspectives. Psychoanalytic orientations theorize that emotions are drive-related, and suppression of these powerful drives is deemed unhealthy (Thomas, 1990). Behavioral orientations conceptualized anger as a reaction to the blocking of a goal (Thomas, 1990). Furthermore, the Skinnerian behavioral conceptualization believes that anger is a learned response to environmental stimuli (Skinner, 1953). Sociocultural theories focus on the interpersonal nature of anger. According to this theory, if the expectations of individuals are not met, it may cause anger (Sullivan, 1953). Contemporary social/psychological perspectives also emphasize that most angry episodes are social events (Thomas, 1990). From the humanistic orientation, emotion is neither an expression of instincts nor a learned response. In the current study, the Teacher Anger Scale (TAS) was employed (Burić and Frenzel, 2019), which was designed based on the above-mentioned theories.

## Materials and methods

### Participants

A sample of 581 teachers (283 male and 298 female) participated in this research, who were teaching English as a foreign language at different private language institutes and at different levels of English proficiency in Iran. To be able to achieve generalizability, the following criteria in selecting the participants were considered: variability in age groups, years of teaching experience, genders, and cities where teachers work. The age range of participants in this phase was between 23 and 53 (*M* = 40.300, *SD* = 9.207) with 1–30 years of teaching experience (*M* = 18.233, *SD* = 9.115). They majored in different branches of English: English Teaching (295), English Literature (94), English Translation (151), and also linguistics (41). Among the participants, 42 teachers were Ph.D. candidates, and 294 teachers held MA degrees; the rest of the teachers were BA.

### Procedure

The data collection for this study started in November 2021 and ended in February 2022 through a web-based platform. That is, the participants received an electronic survey form including the Language Teacher Emotion Regulation Inventory (LTERI), the Teacher Sense of Efficacy Scale (TSES), the ETS, and the TAS through Google Forms. Since all teachers were qualified enough in English, the language of all four scales was English and, in this way, a construct-irrelevant factor was avoided. Conducting the electronic survey enables researchers to collect data from different regions with varying age groups and teaching experiences. Altogether, 581 forms were received with an 84.5% return rate. On account of the design of the electronic survey (each part in the electronic survey form was designed to be necessarily linked), no data were missed.

### Instruments

#### The language teacher emotion regulation inventory

The LTERI (Supplementary Appendix 1), designed and validated by Heydarnejad et al. (2021b), is based on the process model of emotion regulation (Gross, 1998, 2014; Gross and Thompson, 2007) and semi-structured interviews with EFL high school teachers and university professors. This scale requires the language teachers to think about similar situations from their teaching experiences and rate the statements in the light of their employed emotion regulation strategies. The LTERI contains 27 items on a five-point Likert scale anchored by 1 (“never”) and 5 (“always”) with six components, i.e., situation selection, situation modification, attention deployment, reappraisal, suppression, and seeking social support. Heydarnejad et al. (2021b) reported acceptable reliability for all sub-scales of the LTERI (ranging from 0.718 to 0.814). In the present study, the results of Cronbach’s alpha test were acceptable (ranging from 0.715 to 0.801).

#### The teacher sense of efficacy scale

To determine teachers’ self-efficacy beliefs, the Teachers’ Sense of Efficacy Scale (long form) (Supplementary Appendix 2) developed and validated by Tschannen-Moran et al. (1998) was employed. This scale consists of 24 items on a 9-point Likert scale with three sections: (1) efficacy in student engagement, (2) efficacy in instructional strategies, and (3) efficacy in classroom management. The total reliability and reliability of each factor reported by Tschannen-Moran et al. (1998) were satisfactory. In this study, the reliability of the TSES estimated through Cronbach’s alpha was acceptable (ranging from 0.796 to 0.870).

#### The engaged teacher scale

Teachers’ engagement at work was assessed using the Engaged Teacher Scale (ETS) (Supplementary Appendix 3) by Klassen et al. (2013). This instrument includes 16 items, using a seven-point Likert scale (1 = Strongly disagree; 7 = Strongly agree), with four subscales that represent the underlying dimensions of teacher engagement at work: cognitive engagement, emotional engagement, social engagement with students, as well as social engagement with colleagues. In a quantitative study by Azari Noughabi et al. (2020), the reliability of the scale was reported as satisfactory (Cronbach’s alpha = 0.89). In the current study, the reliability of the scale estimated *via* Cronbach’s alpha was acceptable for all sub-components of ETS (ranging from 0.796 to 0.898).

#### The teacher anger scale

To assess teacher anger, the TAS (Supplementary Appendix 4), designed and validated by Burić and Frenzel (2019), was employed. It contains 16 items on a five-point Likert scale (1 = never, 5 = always) and four facets of teacher anger: (1) students-related, (2) parents-related, (3) colleagues-related, and 4) system-related. The reported reliability indices in the study of Burić and Frenzel (2019) were acceptable (ranging from 0.77 to 0.82). In the present study, the reliability of the TAS was estimated *via* Cronbach’s alpha, and the result was satisfactory (ranging from 0.713 to 0.865).

### Data analysis

To analyze the data, CFA and SEM using LISREL 8.80 were conducted. SEM is a robust multivariate procedure used to take a confirmatory hypothesis-testing approach for the proposed structural theory (Schreiber et al., 2006). An SEM model involves two parts: the measurement model and the structural model. The measurement model is used to examine the relationships between the observed variables and latent variables (Hair et al., 1998). The structural model is used to gauge the relationships between the latent variables.

## Results

The results of the statistical analysis computed by the collected data are reported in this section.

The descriptive statistics of EFL in-service teachers**’** emotion regulation strategies, self-efficacy beliefs, work engagement, and anger were displayed in the following table.

Based on Table 1, among language teacher emotion regulation strategies, situation selection (*M* = 4.109, *SD* = 0.672) and situation modification (*M* = 3.806, *SD* = 0.532) got the highest mean scores, whereas the mean score of suppression was the least (*M* = 2.883, *SD* = 0.767). Furthermore, among the components of self-efficacy beliefs, instructional strategies (*M* = 6.941, *SD* = 0.857) displayed the highest mean scores. Student engagement (*M* = 6.835, *SD* = 0.776) and classroom management (*M* = 6.816, *SD* = 0.712) were the subsequent subscales of self-efficacy beliefs endorsed by EFL in-service teachers.

**TABLE 1:** Descriptive statistics.

Inventory	N	Minimum	Maximum	Mean	Std. deviation
Situation selection	581	1.00	5.00	4.109	0.672
Situation modification	581	1.00	4.40	3.806	0.532
Attention deployment	581	1.00	4.25	3.337	0.638
Reappraisal	581	1.00	4.20	3.405	0.586
Suppression	581	1.00	4.00	2.883	0.767
Seeking social support	581	1.50	4.50	3.477	0.654
Student engagement	581	5.00	8.38	6.835	0.776
Instructional strategies	581	4.25	9.00	6.941	0.857
Classroom management	581	5.00	9.00	6.816	0.712
Cognitive engagement	581	3.25	7.00	6.228	0.953
Emotional engagement	581	4.00	7.00	6.361	0.665
Social engagement: students	581	3.50	7.00	6.176	0.806
Social engagement: colleagues	581	2.50	7.00	5.618	0.800
Students-related	581	2.25	5.00	3.296	0.628
Parents-related	581	1.50	5.00	2.401	0.884
Colleagues-related	581	1.75	5.00	2.835	0.834
System-related	581	1.00	5.00	2.704	0.947

Among the subscales of teacher engagement, emotional engagement presented the highest mean scores (*M* = 6.361, *SD* = 0.665). The mean scores of the subsequent sub-scales were as follows: cognitive engagement (*M* = 6.228, *SD* = 0.953), social engagement: students (*M* = 6.176, *SD* = 0.806), and social engagement: colleagues (*M* = 5.618, *SD* = 0.800). Regarding teacher anger subscales, student-related anger (*M* = 3.296, *SD* = 0.628) was the main cause of Iranian EFL in-service teachers’ anger at the workplace. Colleagues-related (*M* = 2.835, *SD* = 0.834), system-related (*M* = 2.704, *SD* = 0.947), and parents-related anger (*M* = 2.401, *SD* = 0.884) were the subsequent triggers of Iranian EFL in-service teachers’ anger at the workplace.

The Kolmogorov-Smirnov Test was utilized to inspect the normality distributions of the data and, consequently, to decide on employing a suitable statistical method for the current study. In the following table, the result of the Kolmogorov-Smirnov test is provided.

As Table 2 displayed, the data were normally distributed because the sig value for all the instruments and their subcomponents was higher than 0.05. To examine the structural relations, the proposed model was tested using the LISREL 8.80 statistical package. To gauge the model fit, the following fit indices were used: the chi-square magnitude, the Root Mean Square Error of Approximation (RMSEA), the comparative fit index (CFI), and the normed fit index (NFI). According to Jöreskog (1990), the chi-square should be non-significant and the chi-square/df ratio should be lower than 3. Also, the root mean square error of approximation (RMSEA) should be lower than 0.1 (Jöreskog, 1990). The NFI with the cut value greater than 0.90, GFI with the cut value greater than 0.90, and CFI with the cut value greater than 0.90 indicates a good fit (Jöreskog, 1990). As Table 3 presents, the chi-square/df ratio (2.833) and the RMSEA (0.056) reached the acceptable fit thresholds. The other three fit indices, GFI (0.972), NFI (0.921), and CFI (0.932) were also acceptable.

**TABLE 2:** The results of Kolmogorov-Smirnov test.

Inventory	Sub-scales	N	Kolmogorov-Smirnov Z	Asymp. Sig. (2-tailed)
The language teacher emotion regulation inventory	Situation selection situation modification attention	581	1.205	0.110
		581	1.315	0.063
	Deployment	581	1.252	0.087
	Reappraisal	581	0.840	0.481
	Suppression	581	1.209	0.107
	Seeking social support	581	0.903	0.388
Total		581	1.081	0.193
The teachers’ sense of efficacy scale	Student engagement	581	1.280	0.075
	Instructional strategies	581	0.570	0.901
	Classroom management	581	0.946	0.332
Total		581	0.881	0.419
The engaged teacher scale	Cognitive engagement	581	0.946	0.333
	Emotional engagement	581	1.320	0.065
	Social engagement: Students	581	1.317	0.061
	Social engagement: Colleagues	581	0.855	0.458
Total		581	1.155	0.139
The Teacher Anger Scale	Students-related	581	0.882	0.417
	Parents-related	581	1.180	0.123
	Colleagues-related	581	0.948	0.330
	System-related	581	0.527	0.944
Total		581	0.892	0.404

**TABLE 3:** Model fit indices.

Fitting indexes	χ^2^	*df*	χ^2^/*df*	RMSEA	GFI	NFI	CFI
Cut value			<3	<0.1	>0.9	>0.9	>0.9
The first model	328.69	116	2.833	0.056	0.972	0.921	0.932

**FIGURE 2:**
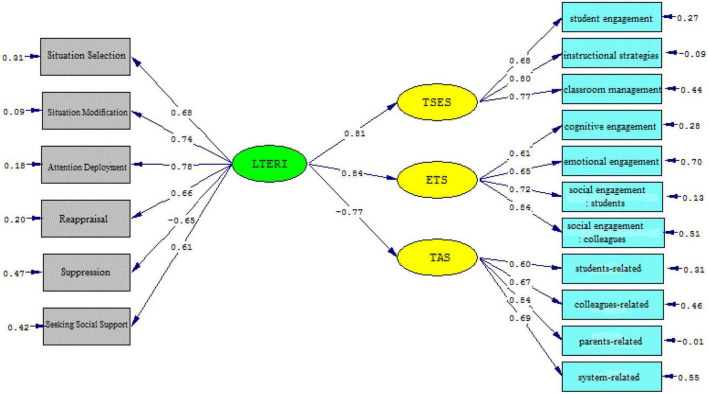
The schematic representation of path coefficient values (Model 1).

**FIGURE 3:**
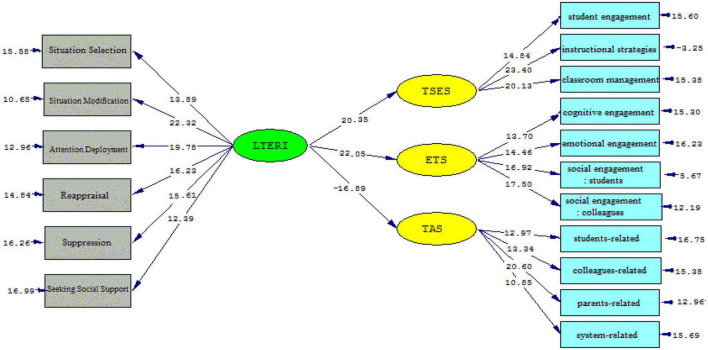
*T*-values for path coefficient significance (Model 1).

The *t*-values and standardized estimates were inspected to gauge the strength of the causal relationships among the variables. As Figures 2, 3 illustrate, language teacher emotion regulation affects teachers’ sense of efficacy beliefs (β = 0.81, *t* = 20.35) and engagement (β = 0.84, *t* = 22.05) significantly and positively; the *t*-value was greater than 1.96. The effect of language teacher emotion regulation on teacher anger was significantly negative (β = –0.77, *t* = –16.89) and the *t*-value was lower than –1.96.

Table 4 also displays the acceptable criteria for fit indices in the second model (phase 4). That is, the chi-square/df ratio (2.730) and the RMSEA (0.055) reached the acceptable fit thresholds. Moreover, GFI (0.912), NFI (0.962), and CFI (0.922) were acceptable.

**TABLE 4:** Model fit indices.

Fitting indexes	χ^2^	*df*	χ^2^/*df*	RMSEA	GFI	NFI	CFI
Cut value			<3	<0.1	>0.9	>0.9	>0.9
The second model	4963.76	1818	2.730	0.055	0.912	0.962	0.922

**FIGURE 4:**
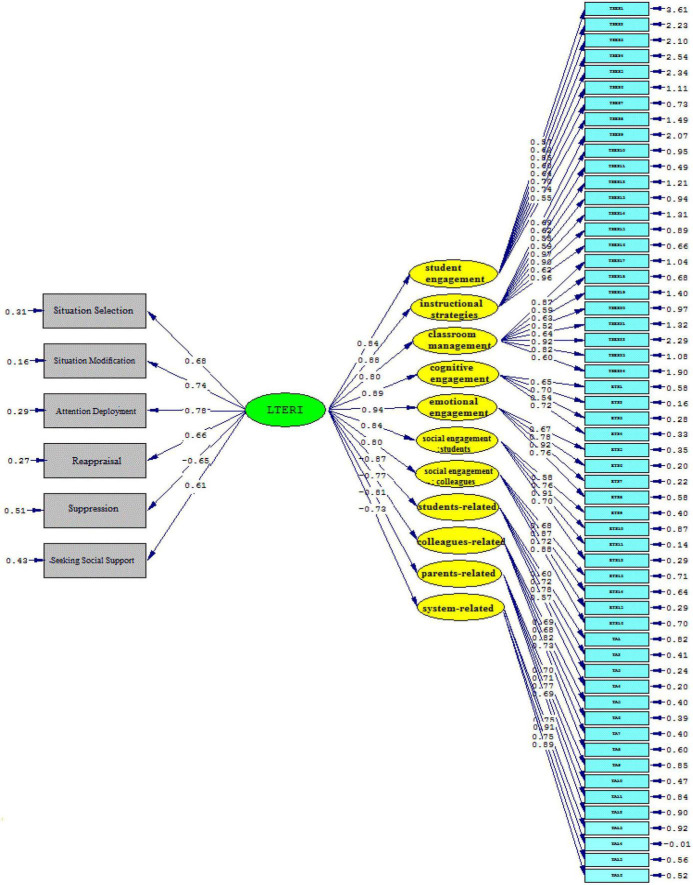
The schematic representation of path coefficient values (Model 2).

**FIGURE 5:**
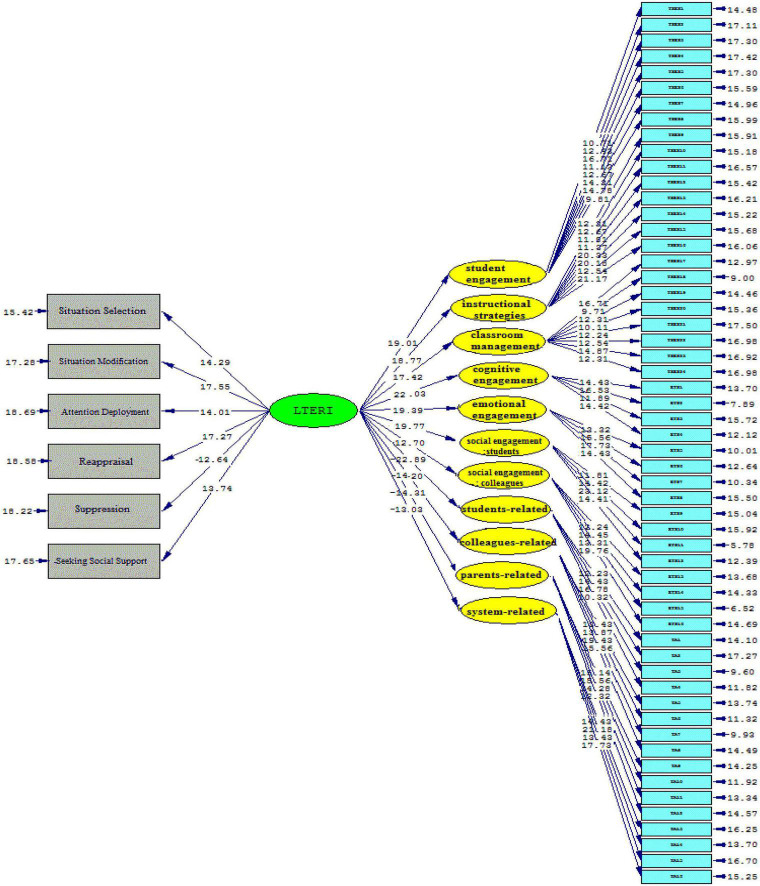
*T*-values for path coefficient significance (Model 2).

Figures 4, 5 (Model 2) demonstrate that language teacher emotion regulation influenced all sub-components of teacher self-efficacy beliefs significantly and positively, as follows: student engagement (β = 0.84, *t* = 19.01), instructional strategies (β = 0.88, *t* = 18.77), classroom management (β = 0.80, *t* = 17.42). The same holds true for the sub-components of the Engaged Teacher Scale. That is, language teacher emotion regulation influenced cognitive engagement (β = 0.89, *t* = 22.03), emotional engagement (β = 0.94, *t* = 19.39), social engagement (students) (β = 0.84, t = 19.77), and social engagement (colleagues) (β = 0.80, *t* = 12.70) significantly and positively. Moreover, the significantly negative effects of language teacher emotion regulation on the sub-components of teacher anger were illustrated as follows: students-related (β = –0.87, *t* = –22.89), parents-related (β = –0.77, *t* = –14.20), colleagues-related (β = –0.81, *t* = –14.31), and system-related (β = –0.73, *t* = –13.03).

A Pearson product-moment correlation was run to examine the relationships among the variables.

As indicated in Table 5, there are significant relationships among the LTERI and student engagement (*r* = 0.619, *p* < 0.01), instructional strategies (*r* = 0.640, *p* < 0.01), and classroom management (*r* = 0.599, *p* < 0.01). Considering the correlations among the LTERI and the sub-components of engaged teacher scale, the results are as follows: cognitive engagement (*r* = 0.461, *p* < 0.01), emotional engagement (*r* = 0.536, *p* < 0.01), social engagement: student (*r* = 0.520, *p* < 0.01), and social engagement: colleagues (*r* = 0.432, *p* < 0.01). In addition, the relationships among the LTERI and the subcategories of the TAS are as follows: students-related (*r* = –0.587, *p* < 0.01), parents-related (*r* = –0.298, *p* < 0.05), colleagues-related (*r* = –0.361, *p* < 0.01), and system-related (*r* = –0.202, *p* < 0.05).

**TABLE 5:** The correlation coefficients among language teacher emotion regulation and the sub-components of self-efficacy beliefs, engagement, and anger.

	LTERI	Student engagement	Instructional strategies	Classroom management	Cognitive engagement	Emotional engagement	Social engagement: students	Social engagement: colleagues	Students-related	Parents-related	Colleagues-related	System-related
LTERI	1											
Student engagement	0.619**	1										
Instructional strategies	0.640**	0.770**	1									
Classroom management	0.599**	0.686**	0.780**	1								
Cognitive engagement	0.461**	0.518**	0.712**	0.593**	1							
Emotional engagement	0.536**	0.425**	0.485**	0.446**	0.466**	1						
Social engagement: students	0.520**	0.414**	0.467**	0.620**	0.530**	0.587**	1					
Social engagement: colleagues	0.432**	0.469**	0.402**	0.668**	0.395**	0.534**	0.511**	1				
Students-related	–0.587**	–0.686**	–0.539**	–0.548**	–0.425**	–0.546**	–0.522**	–0.594**	1			
Parents-related	–0.298*	–0.317*	–0.307*	–0.341**	–0.377**	–0.440**	–0.418**	–0.370**	0.473**	1		
Colleagues-related	–0.361**	–0.385*	–0.437**	–0.507**	–0.288*	–0.359**	–0.417**	–0.333**	0.579**	0.650**	1	
System-related	–0.202*	–0.316*	–0.233*	–0.390**	–0.317*	–0.247**	–0.273*	–0.331**	0.437**	0.501**	0.628**	1

**Correlation is significant at the 0.01 level (2-tailed).

*Correlation is significant at the 0.05 level (2-tailed).

## Discussion

This study was an attempt to uncover the interrelatedness of language teacher emotion regulation, self-efficacy, engagement, and anger. This aim was accomplished by utilizing a structural equation modeling approach targeted at building a causal structural model by which the contribution of each of the aforementioned constructs can be estimated. Data analyses indicated that the EFL teachers’ emotion regulation predicts their self-efficacy skills and engagement in a positive direction. In contrast, the effect of the EFL teachers’ emotion regulation on their feelings of anger is in a negative direction.

Emotion regulation strategies offer teachers ways to control their emotions and act effectively. The more teachers practice emotion regulation, the better they can manage and modify the emotional demands. This capacity offers the self-measurement of teachers’ emotional experiences and gives directions to their professional wellbeing. The findings of the present study revealed a close relationship between the language teachers’ emotion regulation and their self-efficacy belief (see Model 1). Therefore, the first null hypothesis of the study (H01. EFL teachers’ emotion regulation has no impact on their self-efficacy beliefs in the workplace) was rejected. A detailed inspection of the results indicated emotion regulation influences the teacher self-efficacy sub-components (student engagement, instructional strategies, and classroom management) significantly (see Model 2). Scrutinizing the relevant literature on teacher emotion and teacher self-efficacy beliefs echoes a growing body of research that illuminates the intertwined relationship between teacher emotion and their efficacy (Burić et al., 2017; Burić and Macuka, 2017; Burić et al., 2020; Liu H. et al., 2021; Chen and Cheng, 2022). In this regard, Burić and Moè (2020) acknowledged the interrelationships of teacher self-efficacy, positive emotions, and teacher wellbeing, which affect teacher enthusiasm. Similarly, Chen (2018) confirmed that teacher efficacy is positively associated with their emotions. This finding is in accord with those of Fathi et al. (2020), Heydarnejad et al. (2021a), Liu H. et al. (2021), and Amirian et al. (2022) as well as Xiyun et al. (2022). Additionally, Cansoy et al. (2020) and Zheng et al. (2022) confirmed that teacher emotional wellbeing and self-efficacy beliefs are interrelated. According to the LTER model (Heydarnejad et al., 2021b), emotion regulation is the output of appraisal, attention deployment, situation modification, seeking social support, situation selection, and suppression. When teachers use effective emotion regulation strategies, the better they can manage and modify their emotional experiences (Li et al., 2022; Zheng et al., 2022). Effective emotion regulation can affect teachers’ sense of efficacy. Based on Bandura’s self-efficacy theory (Bandura, 1982), efficacious teachers implement mastery experiences and use social experiences for successful achievement. Furthermore, it is implied that the psychological and affective states as sources of teacher self-efficacy can be regulated *via* emotion regulation and help their cognitive development.

The predictive power of language teacher emotion regulation on their work engagement, as another objective of this study, was confirmed by the results of the present study (see Model 1). Thus, the second null hypothesis of the study (H02. EFL teachers’ emotion regulation has no impact on their engagement at the workplace) was rejected. In detail, language teacher emotion regulation significantly and positively influenced cognitive engagement, emotional engagement, social engagement (students), and social engagement (colleagues) (see Model 2). It implies that emotion-regulatory strategies provide a balance in the professional lives of language teachers. In such a situation, language teachers feel more enthusiasm and engagement in teaching activities. From these findings, it can also be inferred that a language teacher’s emotion regulation maximizes social engagement with students and colleagues. Therefore, for teachers, emotion regulation pinpoints the effective path for raising teachers’ social commitment and enhancing their personal and job accomplishments. This result can be supported by the underpinning premises of the LTER model and SDT (Mulyani et al., 2021; Wang et al., 2022). That is, implementing each of the emotion regulatory strategies enhances the emotional balance of teachers’ professional lives and can result in teachers’ engagement. Additionally, this outcome reconciles with prior studies, though limited and quite rare in the EFL context, which focus on the relationship between teachers’ emotions and their engagement in the workplace (Zeng et al., 2019; Lazarides and Warner, 2020; Topchyan and Woehler, 2020; Liu and Chu, 2022).

Moreover, the results of the present study shed more light on the impact of teacher emotion regulation on their anger. As it was concluded, the influence of emotion regulation on teacher anger was significantly negative (see Model 1); thus, the third null hypothesis (H03. EFL school teachers’ emotion regulation has no impact on their anger management at the workplace) was rejected. To be precise, the data analysis indicated that the main cause of Iranian EFL teachers’ display of anger is student-related. Following student-related anger, colleagues-related anger, system-related anger, and parents-related anger, respectively, trigger Iranian EFL teachers’ anger, which necessitates the role of emotion regulation (see Model 2). This outcome seems plausible, both theoretically and experimentally, given that language teachers spend most of their time dealing with their students and then colleagues (Burić and Frenzel, 2019). Teaching is under the influence of teachers’ social relationships with students and colleagues and any inconsistencies in these reciprocal relationships may trigger a language teacher’s anger. In such situations, teachers need to manipulate their expressions of anger and align them with the emotional display rules of their profession. Therefore, one of the overriding influences of emotion regulation is the efficient handling of everyday problems and affairs. Furthermore, it can be implied that language teachers with high emotion regulation are inclined toward playing safe and relinquishing in the face of challenges and difficulties (Liu et al., 2022).

Furthermore, there were an increasing number of sources of teacher anger, particularly during the coronavirus pandemic with its severe disruption of normal everyday life and unpredictability for the future. In such conflicts, emotion regulation helps teachers navigate the contingencies of the workplace and a new set of challenges caused by the coronavirus pandemic, which have been added to the causes of teacher anger in the last 2 years. In the heat of the moment, teachers need to heal the maladaptive anger and aggression, and this can be plausible by practicing emotion regulation strategies (Burić et al., 2017; Chang and Taxer, 2021).

## Conclusion and implications

Overall, the contextual triggers of language emotion regulation and its significant relationship with teacher’s self-efficacy, engagement, and anger in the Iranian EFL context were inferred in the current research. More precisely, the hypothesized predictive power of language teacher emotion regulation in improving the teacher’s self-efficacy, engagement, and control of anger was verified. The implications drawn from the current study assist language teacher educators in developing more productive pre-service and in-service programs by incorporating these findings into their studies. EFL teacher preparation programs should concentrate on teaching helpful strategies to enhance efficacy beliefs, emotion regulation, and reflection for pre-service teachers. Furthermore, policymakers are invited to consider these results in order to have a comprehensive picture of the factors that contribute to the success and failure of teachers and educational programs. Last but not least, the results of this study could be of importance to any of the educational field’s stakeholders, particularly language teaching, as the challenges brought by the Covid-19 pandemic are still not over, and a coherent human resources strategy should continue to be developed and improved. Furthermore, adding the implications of the current research into pre-service and in-service teacher training programs can pave the way for triggering self-aid skills, which are of great help, especially during the global crisis of the COVID-19 pandemic. These programs are anticipated to pinpoint the most effective path for enhancing their effective teaching.

The findings of this study suggest some pedagogical implications for teacher trainers and EFL teachers. Learning more about situational and personality determinants of the effectiveness of specific emotion regulation strategies can be achieved by doing some particular training programs and informing teachers of the importance of emotions. Such training programs should concentrate on practicing the broad repertoire of strategies and showing the conditions under which they are effective or not. Moreover, training should focus on reflecting more on language teachers’ own traits and preferences that may influence the effectiveness of their employed emotion regulation strategies. This also serves to provoke teachers to alter or modify their employed emotion regulation strategies to more positive ones, which are in turn expected to facilitate their effectiveness.

### Limitations and suggestions for further research

This research, as is the case with other studies in the realm of education, has some limitations. Given that this study is one of the first endeavors in EFL literature, it can be deemed as a prelude to initiating other studies. Undoubtedly, the research agenda in this area is essential to capture the broad aspects and issues pertaining to EFL teachers’ effectiveness. Further experimental studies are suggested in which different emotion regulation strategies are experimentally induced and personality traits are measured. For instance, future longitudinal studies are recommended to investigate the long-term effects of applying certain strategies by teachers, in particular language teachers with specific personality characteristics. Additionally, future studies are suggested to explore the mediating role of personality characteristics of teachers on the effectiveness of different strategies in different situations and settings.

If the debate is to be moved forward, future investigations can take more mixed-method approaches to inspect the studied association here. This could be a fruitful endeavor since the present study lacks a qualitative, data-driven conceptualization of teachers’ and educators’ perspectives. In this study, the relationships between teachers’ years of teaching experience, educational levels, age, and gender and their preferred emotion regulation strategies, self-efficacy beliefs, work engagement, and anger management were not considered. Thus, researchers are recommended to consider these factors in similar research studies in the future. Furthermore, it is recommended to undertake further research to investigate whether language teacher emotion regulation affects language learners’ emotion regulation. As a future perspective, the possible interplay among teacher emotion regulation, self-efficacy, engagement, and anger can be inspected in other educational contexts.

## Data availability statement

The original contributions presented in this study are included in the article/Supplementary material, further inquiries can be directed to the corresponding author/s.

## Author contributions

All authors listed have made a substantial, direct, and intellectual contribution to the work, and approved it for publication.
