# Supplementary material for: Delving into the relationship between teacher emotion regulation, self-efficacy, engagement, and anger: A focus on English as a foreign language teachers
Source: Front Psychol. 2022 Oct 19;13:1019984. doi: 10.3389/fpsyg.2022.1019984 (PMC9627275; doi:10.3389/fpsyg.2022.1019984)
Supplement: Supplementary file 1 [file Data_Sheet_1.docx]

**Appendix 1: Language Teacher Emotion Regulation Inventory (LTERI)**

| Gender: | Major: | | Degree: | Age: |
| --- | --- | --- | --- | --- |
| Years of teaching experience: | |  | Place of teaching: |  |

| **Directions:** Please read the following emotion regulation strategies. Consider similar situations from your own teaching experiences and rate the statements on a 5-point scale. Your answers are confidential. Thanks in advance for your cooperation. **(1= Never, 2= Seldom, 3= Sometimes, 4= Often, 5= Always).** | | | | | | |
| --- | --- | --- | --- | --- | --- | --- |
| LTERI Sub-scales | Items | 1 | 2 | 3 | 4 | 5 |
| A. Situation Selection | 1. In my classroom, I avoid conﬂicting or emotionally annoying situations. | 1 | 2 | 3 | 4 | 5 |
|  | 2. I try to evade unpleasant discussions. | 1 | 2 | 3 | 4 | 5 |
|  | 3. I avoid conﬂicting or emotionally disturbing situations in the staff room. | 1 | 2 | 3 | 4 | 5 |
|  | 4. I try to avoid arguing with troublesome parents. | 1 | 2 | 3 | 4 | 5 |
|  | 5. In my work, I try to avoid a certain situation that may bring about undesirable emotions. | 1 | 2 | 3 | 4 | 5 |
| B. Situation Modification | 6. When I feel helpless at work, I think about my teaching methods critically. | 1 | 2 | 3 | 4 | 5 |
|  | **7.** If my students make me angry in language classes, I try to advise them. | 1 | 2 | 3 | 4 | 5 |
|  | 8. When an unpleasant discussion is raised in my classes, I try to change the topic. | 1 | 2 | 3 | 4 | 5 |
|  | 9. As I improve my knowledge and skills, I can react better in stressful situations at work. | 1 | 2 | 3 | 4 | 5 |
|  | 10. When I face an upsetting conversational topic, I try to substitute it with suitable ones. | 1 | 2 | 3 | 4 | 5 |
| C-Attention Deployment | 11. When I feel anxious in my language classes, I shift my attention to something pleasant. | 1 | 2 | 3 | 4 | 5 |
|  | 12. If I feel frustrated in language classes, I try to engage myself in different class activities to forget it. | 1 | 2 | 3 | 4 | 5 |
|  | 13. In language classes, if I feel unhappy, I try to think about something interesting. | 1 | 2 | 3 | 4 | 5 |
|  | 14. When I feel upset in my language classes, I redirect my attention to more pleasant matters. | 1 | 2 | 3 | 4 | 5 |
| D- Reappraisal | 15. I try to reduce the tension experienced in my language classes by reminding myself that there are more important things in my life. | 1 | 2 | 3 | 4 | 5 |
|  | 16. If my students’ misbehavior makes me angry, I remind myself that they are inexperienced. | 1 | 2 | 3 | 4 | 5 |
|  | 17. When I feel ashamed, I remind myself that I can do better in the future. | 1 | 2 | 3 | 4 | 5 |
|  | 18. If for some reasons, I feel upset at work, I remind myself of my goals in my life. | 1 | 2 | 3 | 4 | 5 |
|  | 19. If I feel hopeless at work, I calm myself by viewing things from another perspective. | 1 | 2 | 3 | 4 | 5 |
| E-Suppression | 20. If I feel anxious in my language classes, I try to suppress that. | 1 | 2 | 3 | 4 | 5 |
|  | 21. If I feel helpless in my language classes, I disregard that. | 1 | 2 | 3 | 4 | 5 |
|  | 22. If for some reasons, I feel angry in my language classes, I overlook that. | 1 | 2 | 3 | 4 | 5 |
|  | 23. When I feel unhappy at work, I ignore that. | 1 | 2 | 3 | 4 | 5 |
| F-Seeking Social Support | 24. When I feel frustrated in my language classes, I share my troubles with my colleagues. | 1 | 2 | 3 | 4 | 5 |
|  | 25. When I feel hopeless in my language classes, I seek advice from experts such as psychologists and school counselors. | 1 | 2 | 3 | 4 | 5 |
|  | 26. If I feel nervous in my language classes, I talk about it with someone who can understand me. | 1 | 2 | 3 | 4 | 5 |
|  | 27. To get my mind off an upsetting situation at work, I talk about it with someone who is close to me. | 1 | 2 | 3 | 4 | 5 |

**Appendix 2: Teachers’ Sense of Efficacy Scale (long form)**

| Gender: | Major: | | Degree: | **Age:** |
| --- | --- | --- | --- | --- |
| Years of teaching experience: | |  | Place of teaching: |  |

| **Teacher Beliefs**  **Directions:** This questionnaire is designed to help us gain a better understanding of the kinds of things that create difficulties for teachers in their school activities. Please indicate your opinion about each of the statements below. Your answers are confidential. (**1= Nothing, 3= Very little, 5= Some influence, 7= Quite a bit, 9= A great deal)** |  | | | | | | | | |
| --- | --- | --- | --- | --- | --- | --- | --- | --- | --- |
| 1. How much can you do to get through to the most difficult students? | 1 | 2 | 3 | 4 | 5 | 6 | 7 | 8 | 9 |
| 2. How much can you do to help your students think critically? | 1 | 2 | 3 | 4 | 5 | 6 | 7 | 8 | 9 |
| 3. How much can you do to control disruptive behavior in the classroom? | 1 | 2 | 3 | 4 | 5 | 6 | 7 | 8 | 9 |
| 4. How much can you do to motivate students who show low interest in school work? | 1 | 2 | 3 | 4 | 5 | 6 | 7 | 8 | 9 |
| 5. To what extent can you make your expectations clear about student behavior? | 1 | 2 | 3 | 4 | 5 | 6 | 7 | 8 | 9 |
| 6. How much can you do to get students to believe they can do well in school work? | 1 | 2 | 3 | 4 | 5 | 6 | 7 | 8 | 9 |
| **7.** How well can you respond to difficult questions from your students? | 1 | 2 | 3 | 4 | 5 | 6 | 7 | 8 | 9 |
| 8. How well can you establish routines to keep activities running smoothly? | 1 | 2 | 3 | 4 | 5 | 6 | 7 | 8 | 9 |
| 9. How much can you do to help your students value learning? | 1 | 2 | 3 | 4 | 5 | 6 | 7 | 8 | 9 |
| 10. How much can you gauge student comprehension of what you have taught? | 1 | 2 | 3 | 4 | 5 | 6 | 7 | 8 | 9 |
| 11. To what extent can you craft good questions for your students? | 1 | 2 | 3 | 4 | 5 | 6 | 7 | 8 | 9 |
| 12. How much can you do to foster student creativity? | 1 | 2 | 3 | 4 | 5 | 6 | 7 | 8 | 9 |
| 13. How much can you do to get children to follow classroom rules? | 1 | 2 | 3 | 4 | 5 | 6 | 7 | 8 | 9 |
| 14. How much can you do to improve the understanding of a student who is failing? | 1 | 2 | 3 | 4 | 5 | 6 | 7 | 8 | 9 |
| 15. How much can you do to calm a student who is disruptive or noisy? | 1 | 2 | 3 | 4 | 5 | 6 | 7 | 8 | 9 |
| 16. How well can you establish a classroom management system with each group of students? | 1 | 2 | 3 | 4 | 5 | 6 | 7 | 8 | 9 |
| 17. How much can you do to adjust your lessons to the proper level for individual students? | 1 | 2 | 3 | 4 | 5 | 6 | 7 | 8 | 9 |
| 18. How much can you use a variety of assessment strategies? | 1 | 2 | 3 | 4 | 5 | 6 | 7 | 8 | 9 |
| 19. How well can you keep a few problems students form ruining an entire lesson? | 1 | 2 | 3 | 4 | 5 | 6 | 7 | 8 | 9 |
| 20. To what extent can you provide an alternative explanation for example when students are confused? | 1 | 2 | 3 | 4 | 5 | 6 | 7 | 8 | 9 |
| 21. How well can you respond to defiant students? | 1 | 2 | 3 | 4 | 5 | 6 | 7 | 8 | 9 |
| 22. How much can you assist families in helping their children do well in school? | 1 | 2 | 3 | 4 | 5 | 6 | 7 | 8 | 9 |
| 23. How well can you implement alternative strategies in your classroom? | 1 | 2 | 3 | 4 | 5 | 6 | 7 | 8 | 9 |
| 24. How well can you provide appropriate challenges for very capable students? | 1 | 2 | 3 | 4 | 5 | 6 | 7 | 8 | 9 |

**Appendix 3: The Engaged Teacher Scale (ETS)**

| Gender: | Major: | | Degree: | Age: |
| --- | --- | --- | --- | --- |
| Years of teaching experience: | |  | Place of teaching: |  |

| **Directions:** Please read the following items. Consider similar situations from your own teaching experiences and rate the statements on a 7-point scale. Your answers are confidential. Thanks in advance for your cooperation (**1= Strongly disagree, 2=Disagree, 3=Somewhat disagree, 4 = Neither agree or disagree, 5 =Somewhat agree, 6= Agree, 7= Strongly agree.)** | | | | | | | | |
| --- | --- | --- | --- | --- | --- | --- | --- | --- |
| Items | | 1 | 2 | 3 | 4 | 5 | 6 | **7** |
| 1 | I try my hardest to perform well while teaching. | 1 | 2 | 3 | 4 | 5 | 6 | 7 |
| 2 | While teaching, I really ―throw‖ myself into my work | 1 | 2 | 3 | 4 | 5 | 6 | 7 |
| 3 | While teaching, I pay a lot of attention to my work | 1 | 2 | 3 | 4 | 5 | 6 | 7 |
| 4 | While teaching, I work with intensity | 1 | 2 | 3 | 4 | 5 | 6 | 7 |
| 5 | I am excited about teaching | 1 | 2 | 3 | 4 | 5 | 6 | 7 |
| 6 | I feel happy while teaching | 1 | 2 | 3 | 4 | 5 | 6 | 7 |
| 7 | I love teaching | 1 | 2 | 3 | 4 | 5 | 6 | 7 |
| 8 | I find teaching fun | 1 | 2 | 3 | 4 | 5 | 6 | 7 |
| 9 | In class, I show warmth to my students | 1 | 2 | 3 | 4 | 5 | 6 | 7 |
| 10 | In class, I am aware of my students’ feelings | 1 | 2 | 3 | 4 | 5 | 6 | 7 |
| 11 | In class, I care about the problems of my students | 1 | 2 | 3 | 4 | 5 | 6 | 7 |
| 12 | In class, I am empathetic towards my students | 1 | 2 | 3 | 4 | 5 | 6 | 7 |
| 13 | At school, I connect well with my colleagues | 1 | 2 | 3 | 4 | 5 | 6 | 7 |
| 14 | At school, I am committed to helping my colleagues | 1 | 2 | 3 | 4 | 5 | 6 | 7 |
| 15 | At school, I value the relationships I build with my colleagues | 1 | 2 | 3 | 4 | 5 | 6 | 7 |
| 16 | At school, I care about the problems of my colleagues | 1 | 2 | 3 | 4 | 5 | 6 | 7 |

**Appendix 4: The Teacher Anger Scale (TAS)**

| Gender: | Major: | | Degree: | Age: |
| --- | --- | --- | --- | --- |
| Years of teaching experience: | |  | Place of teaching: |  |

|  | **Directions:** Please read the following strategies. Consider similar situations from your own teaching experiences and rate the statements on a 5-point scale. Your answers are confidential. Thanks in advance for your cooperation. **(1= Never, 2= Seldom, 3= Sometimes, 4= Often, 5= Always).** | | | | | |
| --- | --- | --- | --- | --- | --- | --- |
|  | Items | 1 | 2 | 3 | 4 | 5 |
| 1 | The reactions of some students frustrate me so much that I would rather just quit the job. | 1 | 2 | 3 | 4 | 5 |
| 2 | The frustration I feel while working with students undermines my job motivation. | 1 | 2 | 3 | 4 | 5 |
| 3 | Some students make so angry that my face goes red. | 1 | 2 | 3 | 4 | 5 |
| 4 | I get anger-caused headache from the behavior of some students. | 1 | 2 | 3 | 4 | 5 |
| 5 | I feel resistance and anger with parents who overestimate their children's real capabilities. | 1 | 2 | 3 | 4 | 5 |
| 6 | I feel my pulse speeds up out of anger when a parent tells me how to do my job. | 1 | 2 | 3 | 4 | 5 |
| 7 | I am annoyed by parents who do not want to accept their child's real capabilities. | 1 | 2 | 3 | 4 | 5 |
| 8 | I feel revolted towards parents who seem not to “hear” the teacher. | 1 | 2 | 3 | 4 | 5 |
| 9 | I wish to yell when I just think about the improper behavior of some colleagues. | 1 | 2 | 3 | 4 | 5 |
| 10 | The anger I feel about unequal workloads among the school staff makes me want to quit my job. | 1 | 2 | 3 | 4 | 5 |
| 11 | My heart pounds faster when I think about certain annoying colleagues. | 1 | 2 | 3 | 4 | 5 |
| 12 | Unfair distribution of work tasks gets me so frustrated that I get all sweaty. | 1 | 2 | 3 | 4 | 5 |
| 13 | I get annoyed because educational regulations and laws are prescribed by people who have never worked in the school. | 1 | 2 | 3 | 4 | 5 |
| 14 | It infuriates me to think about how little the government invests in education. | 1 | 2 | 3 | 4 | 5 |
| 15 | I am angry when teaching quality has to suffer from poor material working conditions. | 1 | 2 | 3 | 4 | 5 |
| 16 | Increasing demands, but with no changes in status, salary, and working conditions, make me angry. | 1 | 2 | 3 | 4 | 5 |
